# Type 2 diabetes as a modifier of fibrin clot properties in patients with coronary artery disease

**DOI:** 10.1007/s11239-012-0821-8

**Published:** 2012-10-20

**Authors:** Maciej Bochenek, Jaroslaw Zalewski, Jerzy Sadowski, Anetta Undas

**Affiliations:** 1 The John Paul II Hospital, Kraków, Poland; 2 Institute of Cardiology, Jagiellonian University Medical College, 80 Pradnicka St, 31-202 Kraków, Poland

**Keywords:** Coronary artery disease, Diabetes mellitus, Fibrin clot, Fibrinolysis, Platelet activation

## Abstract

Altered fibrin clot structure has been reported both in patients with coronary artery disease (CAD) and those with type 2 diabetes mellitus (DM2). The aim of the present study was to evaluate plasma fibrin clot permeability and susceptibility to lysis in patients with DM2 and CAD. We studied 132 consecutive CAD patients, including 67 subjects with DM2, scheduled for elective coronary artery bypass grafting surgery. Ex vivo plasma fibrin clot permeability (K_s_) and lysis time (t_50%_) induced by 1 μg/mL recombinant tissue plasminogen activator (tPA), along with plasma levels of plasminogen activator inhibitor-1 (PAI-1), thrombin activatable fibrinolysis inhibitor (TAFI), tPA, von Willebrand factor (vWF), P-selectin, soluble CD40 ligand (sCD40L), were measured. Diabetic and non-diabetic patients did not differ in regard to demographics and remaining cardiovascular risk factors. Concomitant DM2 was associated with higher glucose (+24.3 %, *p* < 0.001), fibrinogen (+9.0 %, *p* = 0.037), PAI-1 (+58.7 %, *p* < 0.001), tPA (+24.0 %, *p* < 0.001) and P-selectin (+12.2 %, *p* < 0.001). Compared with the non-diabetic group, the CAD patients with DM2 had lower K_s_ (-6.1 %, *p* = 0.02) and prolonged t_50%_ (+5.1 %, *p* = 0.04). Multiple regression analysis of the whole study group showed that vWF, PAI-1, fibrinogen and DM2 were the independent predictors of t_50%_ (*R* ^2^ = 0.58, *p* < 0.001), while only vWF was an independent predictor of K_s_ (*R* ^2^ = 0.22, *p* < 0.001). This study indicates that DM2 is potent enough to unfavorably affect plasma fibrin clot characteristics despite abnormal clot phenotype typically observed in CAD. Of note, platelet and endothelial markers appear to contribute to fibrin clot properties in CAD concomitant with DM2.

## Introduction

Type 2 diabetes mellitus (DM2) is an independent risk factor of coronary artery disease (CAD) and increases the risk of CAD two to three times [1]. Up to 21.4 % of patients with CAD has DM2 [2]. Moreover, the coexistence of DM2 and CAD impairs cardiovascular prognosis and increases the risk of ischemic events [3, 4]. This observation can be explained by several mechanisms, including concomitant risk factors such as arterial hypertension, obesity, and metabolic abnormalities such as hyperglycemia, hyperinsulinemia, insulin resistance and dyslipidemia, which are responsible for endothelial dysfunction [5]. It has been demonstrated that atherosclerotic plaques in DM2 patients are more prone to rupture [6].

Both DM2 as well as CAD have been reported to unfavorably affect structural and functional characteristics of a fibrin clot relatively resistant to mechanical and enzymatic degradation [7–9]. Fibrin clot structure and functions depend on several genetic and environmental factors present in both clinical entities, including enhanced inflammatory status and oxidative stress [10]. Altered fibrin clot architecture and function both in CAD and DM2 are characterized by lower fibrin clot permeability resulting from a smaller pore size in the fiber network, along with impaired susceptibility to lysis [7–12]. Specifically, the altered fibrin structure in DM2 is largely attributed to glycation of fibrinogen and fibrin [9], which may interfere with fibrin polymerization, cross-linking by FXIII, tissue plasminogen activator (tPA) and plasminogen binding and plasminogen to plasmin conversion [8]. However, the data on this association are not consistent [13]. Moreover, fibrinogen is the main determinant of fibrin clot characteristics and increased fibrinogen levels are commonly observed in DM2. This abnormality results in formation of more compact fibrin clots [13]. Hyperfibrinogenemia has also been reported in several cohorts of CAD patients free of DM2 and implicated in a higher risk of myocardial infarction (MI) [14–16]. Importantly, there is evidence that fibrin is a consistent component of atherosclerotic plaques, and that the presence of fibrin can promote their growth, being involved in the progression of atherosclerosis [17].

It is unclear whether DM2 is a modulator of fibrin clot phenotype which is potent enough to alter the fibrin variables modified already by a number of prothrombotic mechanisms that operate in advanced CAD. It has been demonstrated that acute hyperglycemia observed in up to 50 % of acute MI patients could significantly reduce the efficiency of fibrinolysis but had no effect on clot permeability [9]. In advanced atherosclerosis, the effect of DM2 on fibrin clot properties appeared to be weaker and overruled by the impact of other factors, including hyperhomocysteinemia and elevated C-reactive protein [18]. Despite the fact that more tightly packed and less porous fibrin structure has been observed more often in CAD patients who experienced stent thrombosis in the past, the effect of DM2 on clot properties in this population was negligible [19]. On the other hand, treatment with insulin or biguanide has been shown to make fibrin more permeable [20, 21]. Similarly, statin treatment can improve fibrin clot characteristics in subjects with increased cardiovascular risk [9, 22].

The aim of this study was to evaluate the fibrin clot permeability and susceptibility to lysis in CAD patients with concomitant DM2 and determine factors that may account for alterations in fibrin clot properties in such high-risk population.

## Methods

In this case–control study, we recruited 67 consecutive white patients (44 males and 23 females) with CAD (angiographically documented ≥70 % stenosis in at least one epicardial coronary artery) and documented DM2, scheduled for elective coronary artery bypass grafting surgery (CABG). Diabetes was defined as a history of diabetes regardless of disease duration, need for hypoglycemic agents, or fasting plasma glucose ≥126 mg/dL (7 mmol/L) on two separate occasions. Exclusion criteria were: cancer, acute illness, atrial fibrillation, liver injury, acute coronary syndrome within the previous 6 weeks. Medications were administered in unchanged doses for at least 2 weeks before surgery. A control group comprised 65 non-diabetic CAD patients (49 males and 16 females), scheduled for CABG at the same time and matched for age, gender, and smoking habits. All patients signed an informed consent. The study was approved by the Ethics Committee of the Jagiellonian University.

All subjects calculated an individual body mass index (BMI) using the standard formula: body mass in kilograms divided by the square of height in meters.

Blood samples were drawn from an antecubital vein with a minimal stasis prior to surgery. Lipid profile and glucose was determined by routine laboratory methods. Fibrinogen was determined using the von Clauss method. High-sensitivity C-reactive protein (CRP) was measured by nephelometry (Dade Behring). Commercially available immunoenzymatic assays were used to determine fibrinolytic proteins, including plasma plasminogen activator inhibitor-1 antigen (PAI-1; American Diagnostica, Stamford, CT, USA), tPA (Diagnostica Stago, Asnieres, France), thrombin activatable fibrinolysis inhibitor antigen (TAFI; Chromogenix, Lexington, Massachusetts, United States), von Willebrand factor (vWF; Diagnostica Stago, Asnieres, France), and also platelet activation markers, soluble CD40 ligand (sCD40L; R&D Systems, Indianapolis, IN, USA), and P-selectin (R&D Systems). All the intra-assay and inter-assay coefficients of variation for the ELISA measurements were below 7 %.

Fibrin clot permeability, expressed as a permeation coefficient (K_s_), which indicates the pore size, was determined as described [22]. Clot lysis time was determined using a turbidity assay with slight modifications [22]. Briefly, citrated plasma was diluted with the Tris buffer, containing 20 mmol/L CaCl_2_, 1 U/mL human thrombin (Sigma) and 1 μg/mL recombinant tPA (Boerhinger Ingelheim, Ingelheim, Germany). The time required for a 50 % decrease in clot turbidity (t_50%_) was determined [22]. All measurements were performed by technicians blinded to the sample status. The coefficients of intra- and inter-assay variations were 5–6 %.

### Statistical analysis

The study was powered to have a 80 % chance of detecting a 5 % difference in plasma clot permeability and lysis time using a *p* value of 0.05. In order to demonstrate such a difference or greater, 52 patients were required in each group. For a *p* value of 0.01, 77 patients per group were required.

Continuous variables are expressed as mean ± SD or median (interquartile range) and categorical variables as number (percentage). Continuous variables were checked for normal distribution with Shapiro–Wilk test and compared by Student’s *t* test or the Mann–Whitney *U* test as appropriate. The Pearson or Spearman rank correlation coefficients were calculated to test the association between two variables with a normal or non-normal distribution, respectively. The influence of different therapy of diabetes on fibrin clot properties was checked with Kruskal–Wallis ANOVA with multiple comparisons test. All clinical and laboratory variables that showed the association with Ks or t_50%_ in univariate model (*p* ≤ 0.2) and did not show substantial correlations (*r* > 0.5) with another independent variable were then included in the multiple linear regression analysis to determine predictors of K_s_ and t_50%_ in the study population. A *p* value < 0.05 was considered statistically significant.

## Results

As shown in Table 1, diabetic and non-diabetic groups were similar in terms of demographic variables and cardiovascular risk factors except BMI, which was 9 % higher in the former group (*p* < 0.001). A larger proportion of diabetic patients received angiotensin converting enzyme inhibitors (ACEI; *p* = 0.001). Twenty-six patients in the diabetic group received insulin alone, 27 were on oral hypoglycemic drugs only, 7 received both insulin and oral agents, while 7 patients were only on the diet.

**Table 1:** Patient characteristics

	Diabetic subjects *n* = 67	Controls *n* = 65	*p* value
Age (years)	65.6 ± 7.8	65.3 ± 9.6	0.86
Male gender, *n* (%)	44 (66)	49 (75)	0.22
BMI (kg/m^2^)	29.6 ± 4.0	27.1 ± 3.8	0.002
Arterial hypertension, *n* (%)	61 (91)	54 (83)	0.17
Smoking, *n* (%)	17 (25)	14 (22)	0.60
Peripheral artery disease, *n* (%)	6 (9)	13 (20)	0.07
Previous MI, *n* (%)	53 (79)	55 (87)	0.41
Aspirin, *n* (%)	11 (16)	10 (15)	0.87
Statins, *n* (%)	58 (87)	59 (91)	0.33
Fibrates, *n* (%)	1 (1)	0	0.32
Thienopyridine, *n* (%)	3 (4)	1 (2)	0.32
ACEI, *n* (%)	65 (97)	51 (78)	0.001
β-blocker, *n* (%)	61 (91)	58 (89)	0.73

Data are shown as mean ± SD or median (IQR) unless otherwise indicated

*BMI* body mass index, *MI* myocardial infarction, *ACEI* angiotensin converting enzyme inhibitor

As expected, diabetic patients had higher fasting glucose (+24.3 %, *p* < 0.001) and fibrinogen levels (+9.0 %, *p* = 0.037). Elevated PAI-1 (+58.7, *p* < 0.001), tPA (+24.0 %, *p* < 0.001) antigens, but not TAFI, were observed in the diabetic group. Interestingly, P-selectin was increased (+12.2 %, *p* < 0.001) in the diabetic group. vWF and sCD40L were similar in both groups (Table 2).

**Table 2:** Laboratory investigations

	Diabetic subjects *n* = 67	Control subjects *n* = 65	*p* value
Glucose (mmol/l)	6.8 (5.6–8.0)	5.3 (4.8–5.6)	<0.001
Total cholesterol (mmol/l)	4.91 (4.18–5.39)	4.53 (3.89–5.41)	0.40
Triglycerides (mmol/l)	1.55 (1.21–1.96)	1.48 (1.10–1.79)	0.25
HDL cholesterol (mmol/l)	1.24 (1.09–1.48)	1.31 (1.12–1.52)	0.29
LDL cholesterol(mmol/l)	2.89 (2.42–3.64)	2.75 (2.32–3.57)	0.33
Platelets (×10^3^/mm^3^)	213 (177–248)	219 (179–253)	0.69
Creatinine (μmol/L)	77 (43–172)	84 (43–154)	0.42
CRP (mg/l)	2.60 (1.65–4.41)	2.34 (1.64–4.33)	0.28
Fibrinogen (g/l)	4.43 (3.45–4.96)	3.87 (3.18–4.82)	0.037
PAI-1 (ng/ml)	65.4 (47.9–78.0)	27.0 (21.4–35.0)	<0.001
TAFI (%)	103 (93–117)	106 (99–113)	0.76
tPA (ng/ml)	10.4 (9.0–11.1)	7.9 (6.5–9.9)	<0.001
vWF (IU/ml)	108 (100–129)	108.0 (100–119)	0.31
sCD40L (ng/ml)	1.04 ± 0.24	0.97 ± 0.27	0.10
P-selectin (ng/ml)	199.4 ± 34.3	175.1 ± 39.8	<0.001
K_s_ (10^−9^cm^2^)	7.70 ± 1.34	8.20 ± 0.92	0.02
t_50%_ (min)	9.42 ± 1.47	8.94 ± 1.23	0.04

Data are shown as X ± SD or median (IQR)

*HDL* high-density lipoprotein, *LDL* low-density lipoprotein, *CRP* C-reactive protein, *PAI-1* plasminogen activator inhibitor-1, *TAFI* thrombin activatable fibrinolysis inhibitor, *tPA* tissue plasminogen activator, *vWF* von Willebrand factor, *sCD40L* soluble CD40 ligand, *K*_*s*_ permeation coefficient, *t*_*50%*_ clot lysis time

**Fig. 1:**
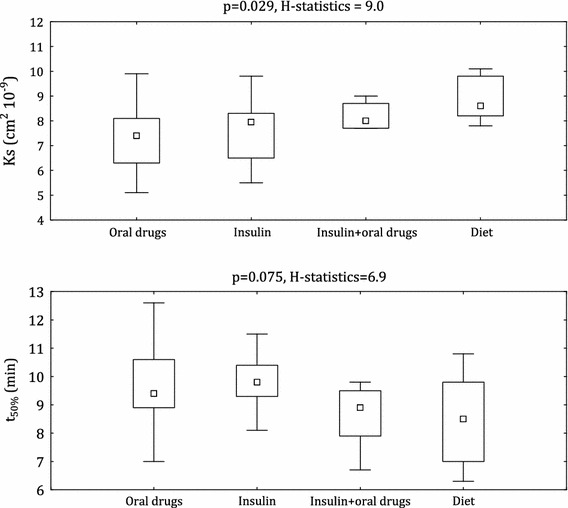
Clot lysis time (t_50%_) and permeation coefficient (K_s_) in relation to different therapy of diabetes. Abbreviations: box plot shows median and interquartile range (IQR) (Q3 to Q1). Q1 and Q3 are the first and third quartiles. Whiskers are drawn at Q3 + 1.5 × IQR, Q1 − 1.5 × IQR. Extreme values are omitted

CAD patients with DM2 had different fibrin clot parameters compared with the non-diabetic controls. K_s_ was 6.1 % lower in the diabetic group (*p* = 0.02) while t_50%_ was 5.1 % longer in patients with DM2 (*p* = 0.04). There were no significant differences in t_50%_ between patients receiving various hypoglycemic therapy (*p* = 0.10) (Fig. 1). However, patients treated with oral hypoglycemic drugs had 16.9 % lower K_s_ than those treated with diet only (*p* < 0.02) and subjects receiving insulin had 14.6 % lower K_s_ than those on dietary treatment only (*p* < 0.09) (Fig. 1).

**Fig. 2:**
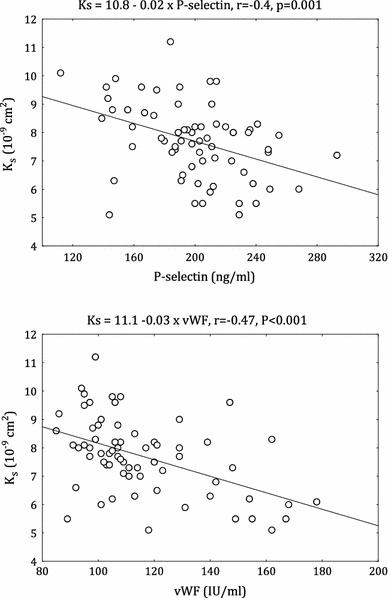
Correlation between permeation coefficient (K_s_) and von Willebrand factor (vWF) and P-selectin in diabetic patients

**Fig. 3:**
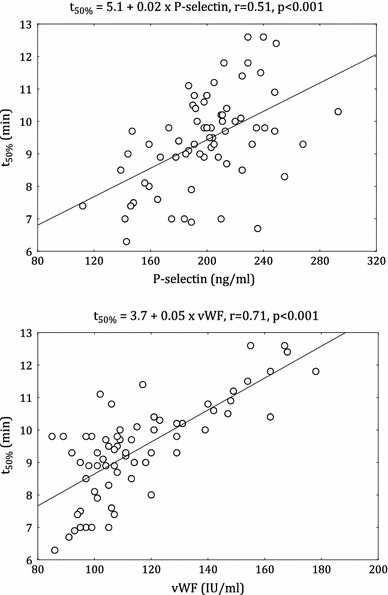
Correlation between clot lysis time (t_50%_) and von Willebrand factor (vWF) and P-selectin in diabetic patients

In diabetic group the inverse associations between K_s_ and TAFI (*r* = −0.36, *p* = 0.003), PAI-1 (*r* = −0.38, *p* = 0.001), tPA (*r* = −0.27, *p* = 0.03) were observed (Table 2). There were also negative correlations between K_s_ and P-selectin (*r* = −0.40, *p* = 0.001; Fig. 3) and vWF (*r* = −0.47, *p* < 0.001; Fig. 2). Moreover, sCD40L and K_s_ (*r* = −0.24, *p* = 0.06) tended to show inverse correlation in the diabetic group. Similar correlations were not seen in the non-diabetic CAD patients (data not shown).

In patients with DM2 but not in the non-diabetic group, there was a positive correlation between t_50%_ and CRP (*r* = 0.31, p = 0.01). The positive associations t_50%_ with TAFI (*r* = 0.53, *p* < 0.001) and tPA (*r* = 0.37, *p* = 0.002) were demonstrated. In addition, PAI-1 had also positive associations with K_s_ in diabetic (*r* = 0.57, *p* < 0.001) and non-diabetic patients (*r* = 0.62, *p* < 0.001). In the both groups, fibrinogen, P-selectin, vWF were positively correlated with t_50%_ (diabetic group (Fig. 3): *r* = 0.41, *p* < 0.001; *r* = 0.51, *p* < 0.001; *r* = 0.71, *p* < 0.001, respectively; non-diabetic group: *r* = 0.52, *p* < 0.001; *r* = 0.36, *p* = 0.003; *r* = 0.49, *p* < 0.001, respectively). Only in the control group, sCD40L was correlated with t_50%_ (*r* = 0.26, *p* = 0.037).

A multiple linear regression analysis was performed to determine the independent effect of laboratory and clinical variables on K_s_ and t_50%_. Before the inclusion to the multiple model, significant correlations between independent variables have been found. K_s_ was strongly correlated with t_50%_ (*r* = −0.67; *p* < 0.001), vWF (*r* = −0.43; *p* < 0.001), PAI-1 (*r* = −0.37; *p* < 0.001), and P-selectin (*r* = −0.29; *p* = 0.001). t_50%_ was significantly correlated with vWF (*r* = 0.64; *p* < 0.001), PAI-1 (*r* = 0.49; *p* < 0.001), P-selectin (*r* = 0.46; *p* < 0.001), fibrinogen (*r* = 0.46; *p* < 0.001), TAFI (*r* = 0.39; *p* < 0.001), and CRP before CABG (*r* = 0.30; *p* < 0.001). P-selectin was correlated with PAI-1 (*r* = 0.47; *p* < 0.001), and fibrinogen (*r* = 0.29; *p* = 0.001) whereas PAI-1 with t-PA (*r* = 0.38; *p* < 0.001), and vWF (*r* = 0.44; *p* < 0.001). Fibrinogen was correlated with CRP (*r* = 0.53; *p* < 0.001), platelet count (*r* = 0.37; *p* < 0.001), and vWF (*r* = 0.30; *p* < 0.001). Finally, the multivariate model for the whole study population showed that vWF, PAI-1, fibrinogen and diabetes mellitus were the independent predictors of t_50%_ (*R* ^2^ = 0.58, *p* < 0.001; Table 3) whereas vWF was the only independent predictor of K_s_ (*R* ^2^ = 0.22, *p* < 0.001; Table 3).

**Table 3:** Multiple linear regression with permeation coefficient or lysis time as the dependent variable

Dependent variable	Independent variable	Contribution of variance, %	*p*	β
K_s_	vWF	10.5	<0.0001	−0.36
	Diabetes mellitus	2.1	0.07	−0.15
	TAFI	1.2	0.10	−0.13
	ACEI	0.04	0.85	−0.02
	CRP	0.01	0.95	−0.01
t_50%_	vWF	16.4	<0.0001	0.43
	PAI-1	16.3	<0.0001	0.45
	Fibrinogen	5.5	0.0004	0.23
	Diabetes mellitus	9.1	0.002	−0.30
	ACEI	0.36	0.33	0.06

*Abbreviations*: see Tables 1 and 2

## Discussion

The present study shows that DM2 is associated with decreased clot permeability and susceptibility to lysis in patients with severe CAD. A strong modulatory effect of DM2 on plasma fibrin clot phenotype has been observed in the presence of several factors leading also to the formation of more dense and less lysable clots such as smoking, hypertension and elevated CRP levels [10].

Dunn et al. [8] have reported that the fibrin clots formed from fibrinogen obtained from patients with DM2 had denser, less porous structure than controls. That was related to glycemic control but they had excluded CAD patients. The lack of associations between both fibrin variables and glycemia indicates that other factors may account for alterations in fibrin clot phenotype in CAD patients.

Platelet activation is a well known modifier of fibrin clot properties. It has been shown that altered clot properties can be caused by a number of platelet-derived proteins mainly released at the sites of platelet aggregation [23]. Polyphosphates secreted from dense granules can alter the formation of fiber aggregates [24]. In the presence of activated platelets, a fibrin clot may have a different architecture with local increase in fiber density [25]. Moreover, it has been demonstrated that the use of antiplatelet agents in patients with DM2 can overcome to some extent the potential effects of poor glycemic control on platelet reactivity [26]. Our study shows that P-selectin, which is expressed in alpha-granules of activated platelets, was increased in patients with DM2 and CAD compared with non-diabetic subjects. Another platelet marker, sCD40L, which did not differentiate diabetic and non-diabetic CAD patients, showed weaker or no associations with fibrin parameters in the current study. This provides evidence for a role of platelet activation in altering the fibrin clot structure in diabetic CAD patients.

An original finding is a strong association between vWF and clot properties, both permeability and lysis, observed in advanced CAD. Interestingly, in this patient group vWF was a predictor of K_s_, indicating novel associations linking endothelial dysfunction with fibrin clot properties measured ex vivo. Endothelial dysfunction implies an alteration in endothelial integrity and, as such, may be assessed by flow-mediated dilatation or by changes in circulating markers, such as plasma vWF [27]. It remains to be established whether vWF per se might actively alter plasma fibrin network properties.

TAFI combines fibrinolysis with coagulation cascade [28]. There have been previous studies showing increased [29–31] or decreased TAFI levels [32, 33] in CAD patients. There have been, however, few reports evaluating the effect of TAFI on clot lysis time in diabetic subjects that demonstrated no effect [34] or hypofibrinolysis [35]. In the present study, despite similar TAFI levels in both groups, we found associations between permeability, lysis time and TAFI only in the diabetic CAD group. The impact of TAFI on plasma fibrin clot phenotype in DM2 merits further investigation.

Given the fact that in both groups, a significant percentage of CAD patients was taking statins, which have been reported to increase the clot permeability and lysis time [9, 22], it might be concluded that both drugs, though they can improve clot phenotype, were not able to abolish a complex effect of DM2 on fibrin variables [9, 11, 13]. Of note, there were differences in clot permeability associated with the mode of treatment of DM2. Taking into account the previous findings [20, 21], this probably resulted from disease severity and a specific effect of insulin or oral antidiabetic agents.

In the present study, PAI-1, fibrinogen and DM2 have been found to be the independent predictors of t_50%_, and vWF both t_50%_ and K_s_. PAI-1 is protein mostly released from the endothelium and from blood platelets. It is responsible for impaired fibrin clot degradation in platelet-rich plasma. It appears that coexistence of atherosclerosis and DM2 might markedly enhance influence of the endothelial cell and the platelet activation on t_50%_. This surprising finding deserves further investigation.

Several limitations of our study warrant consideration. First, the size of this study is limited. Nevertheless, diabetic patients were matched with the non-diabetic group for most parameters. Second, glycated hemoglobin HbA1c was not determined. Third, imaging analysis using scanning electron microscopy was not performed. Nevertheless, sample preparation including dehydratation hampers the extrapolation of microscopic findings to the in vivo conditions.

In conclusion, our observations indicate that the DM2 is potent enough to unfavorably affect the plasma fibrin clot characteristics despite the altered clot phenotype as typically observed in advanced CAD. In this clinical study platelet and endothelial markers appear to contribute to fibrin clot properties in CAD concomitant with DM2. Further studies are needed to determine the mechanism of the altered fibrin clot structure formation in patients with DM2 and advanced CAD.
